# Mitochondrial DNA control region variation in a population sample from Thailand

**DOI:** 10.1007/s00414-020-02303-2

**Published:** 2020-05-01

**Authors:** Dirk Christopher Sauer, Jana Naue, Uta-Dorothee Immel, Sabine Lutz-Bonengel

**Affiliations:** 1 Institute of Forensic Medicine, Medical Center – University of Freiburg, Forensic Molecular Biology, Albertstrasse 9, 79104 Freiburg, Germany; 2 Faculty of Medicine, University of Freiburg, Freiburg, Germany; 3 Institute of Legal Medicine, Johannes Gutenberg-University Mainz, Am Pulverturm 3, 55131 Mainz, Germany; 4 Institute of Legal Medicine, Martin-Luther-University Halle-Wittenberg, Franzosenweg 1, 06114 Halle, Germany

**Keywords:** Mitochondrial DNA, Control region, Thailand, Haplogroup, Forensic database

## Abstract

Mitochondrial DNA (mtDNA) control region sequences from hair samples of 213 individuals from Thailand were analyzed using Sanger sequencing. A total of 170 different haplotypes were identified, of which 146 occurred only once (unique haplotypes). The dataset showed a random match probability of 0.87% and a haplotype diversity of 0.9960. The samples were assigned to 85 different haplogroups with B5a, F1a1a, and M being the most frequent ones. Pairwise F_ST_-values between this and other Southeast and East Asian populations revealed significant but relatively low differences, indicating a close relation. Heteroplasmic positions were observed in 12.2% of hair samples confirming the frequent appearance of heteroplasmic positions in hairs. This dataset will complement existing data as an mtDNA reference for forensic investigations.

**Electronic supplementary material:**

The online version of this article (10.1007/s00414-020-02303-2) contains supplementary material, which is available to authorized users.

## Introduction

Mitochondrial DNA (mtDNA) analysis has become a routine approach in forensic casework where STR markers cannot be used. The associated estimation of the frequency of obtained mtDNA profiles in the respective population sample is based on the availability of suitable population data sets. MtDNA data from Thailand has already been published [1–5]. However, these datasets either are limited to SNPs in the mitochondrial hypervariable regions 1 and 2 [1] or the collection strategy focuses on a different priority, such as language [2–4]. Furthermore, a forensic study on a population from Thailand was done in the northern province of Chiang Mai [5]. Given that the population of Thailand is composed of different ethnolinguistic groups [6], a regional population sample cannot be considered to be representative for the whole country. In this paper, we present a population dataset of 213 individuals living in all four major regions of Thailand.

## Materials and methods

### Samples

**Fig. 1:**
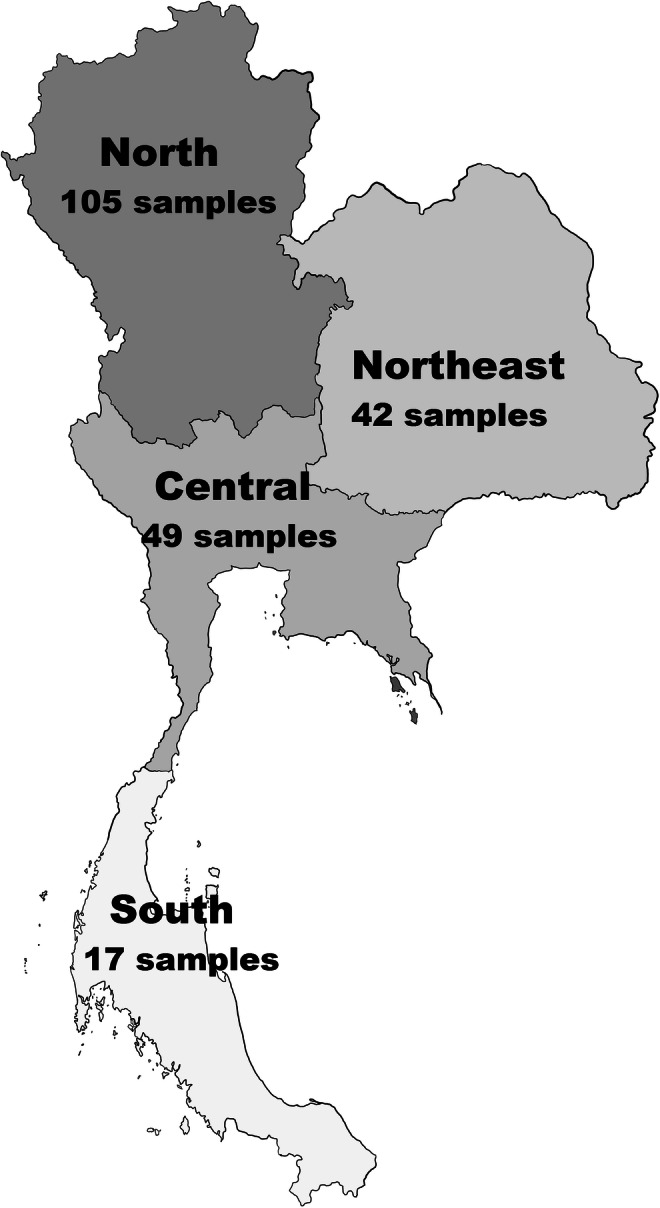
Schematic map of Thailand. The four major regions of Thailand and number of collected samples from each region are provided

Hair samples were obtained from 213 unrelated individuals of both sexes living in southern, central, northern, and northeastern Thailand (Fig. 1, Supplementary Table S1). The samples were collected from volunteer donors and anonymized. Written informed consent was obtained from all participants. Ethical approval for mtDNA sequencing analysis was given by the Ethics Committee of the University of Freiburg, Germany (398/16).

### DNA extraction, amplification, and sequencing

Total DNA of 10–12 hairs per individual was extracted with the MagCore® Genomic DNA Tissue Kit (RBC Bioscience, New Taipei City, Taiwan) at the University of Khon Kaen (Khon Kaen, Thailand). PCR and sequencing of the entire control region were performed at the University Medical Center Freiburg – University of Freiburg (Freiburg, Germany) as described in [7] using primers given in Supplementary Tables S2 and S3.

### Sequence analysis and haplogroup assignment

A total of 213 full double strand control region sequences were successfully analyzed and aligned to the revised Cambridge Reference Sequence (rCRS) [8, 9] using Sequencher V5.2.2 (GeneCodes, Ann Arbor, MI, USA). Alignment was done according to the revised and extended guidelines of the ISFG [10]. Haplogroups were assigned with Haplogrep 2 (www.haplogrep.uibk.ac.at) [11] and EMPOP v4/R12 (www.empop.online) [12] based on PhyloTree, build 17 [13]. Assignments were revised manually and conservative estimates of most recent common ancestors (MRCA) were chosen.

MtDNA data quality was controlled using the EMPOP tool NETWORK [14]. Further quality control was done by the team of EMPOP at Medical University of Innsbruck. All 213 sequences are incorporated into the EMPOP database under the accession number EMP00699.

### Statistical analysis

Intra- and inter-population statistical analysis was done using Arlequin v3.5.2.2 [15]. Numbers of different and unique haplotypes were counted and genetic diversity indices of the population (random match probability, haplotype diversity, number of polymorphic positions, mean number of pairwise differences, and nucleotide diversity) were calculated. Length variants at nucleotide positions 16193, 309, and 573 were ignored for statistical tests. Random match probability was calculated as the sum of squared haplotype frequencies.

We compared our data with six other Southeast and East Asian populations from recent studies [5, 16–20] only considering control region data (np 16024–576). We performed a molecular variance analysis (AMOVA) and calculated genetic diversity indices for the additional included studies, pairwise differences between and within populations, and pairwise F_ST_-values.

## Results and discussion

We obtained 213 high-quality mtDNA control region sequences from Thailand to establish reference data (Supplementary Table S1). Summary statistics are presented in Table 1. From a total of 170 different haplotypes, 146 were unique. The population sample had a random match probability of 0.87% and a haplotype diversity of 0.9960 ± 0.0013 revealing a high heterogeneity in the population making it useful for forensic analyses.

**Table 1:** Molecular diversity indices for mtDNA control region data of a population sample from Thailand

Population statistics*	Thailand (*n* = 213)
Number of different haplotypes	170
Unique haplotypes; portion of samples	146 (68.5%)
Random match probability	0.87%
Haplotype diversity	0.9960 ± 0.0013
Number of polymorphic positions (including indels)	196 of 1134
Mean number of pairwise differences	12.86 ± 5.82
Nucleotide diversity	0.011337 ± 0.005674

*Insertions at nucleotide positions 16193, 309, and 573 were disregarded

We compared the detected haplotypes with those of six earlier studies of Southeast and East Asian populations including one with samples from Northern Thailand [5, 16–20]. A total of 44 of the 170 haplotypes (25.9%) were found in at least one other population (Supplementary Table S4). Accordingly, 126 haplotypes (74.1%) of our study were not observed in the other studies, including the most common haplotype of our study (9 samples) (cf. Supplementary Table S1).

### Haplogroup composition

The 213 samples from Thailand were assigned to 85 different haplogroups (Supplementary Table S1). Some sequences could not be classified to a terminal branch of the PhyloTree [13] and were assigned to their MRCA such as macrohaplogroup M (8.9%). The most frequent terminally assigned haplogroups were B5a (9.4%), F1a1a (8.9%), and M (8.9%). All samples belong to macrohaplogroups R (50.7%), M (39.4%), and N (9.4%) except of one sample which could only be assigned to L3 as MRCA.

In general, the haplogroup composition is comparable with other Southeast Asian population samples with F1, B5, and M being the most frequent lineages [2, 4, 5, 16–19].

### Genetic distances between Southeast and East Asian populations

We compared the genetic structures of our population sample from Thailand with the six other Southeast and East Asian population samples [5, 16–20]. The total number of samples was 1789. Analysis of molecular variance (AMOVA) revealed that 98.06% of the genetic variation is due to differences within populations. Thus, only 1.94% of the total genetic variance is caused by differences between populations (Table 2). Bodner et al. (2011) had found an inter-population variance of only 0.84% in a very similar dataset, but only considering hypervariable regions (HVS-I and HVS-II) and a regional restricted population [16].

**Table 2:** Analysis of molecular variance (AMOVA) of 7 Southeast and East Asian populations (analyzed range: np 16024–576). AMOVA design and results

Source of variation	Degrees of freedom	Sum of squares	Variance components	Percentage of variation
Among populations	6	229.238	0.12603^b^	1.94^a,b^
Within populations	1782	11,375.467	6.38354	98.06
Total	1788	11,604.705	6.50957	

^a^Corresponds to F_ST_ of 0.0194

^b^*p* value < 0.001, number of permutations: 1023

The number of mean pairwise differences (MPD) in the Thai population is 12.86, which is in the dimension of that observed in other Asian populations (Table 3). The lowest MPD value (11.75) was observed in a South Korean population, the highest (13.42) in Northern Thailand.

**Table 3:** Analysis of molecular variance (AMOVA) of 7 Southeast and East Asian populations (analyzed range: np 16024–576). Mean number of pairwise differences (MPD) between seven Southeast and East Asian populations

Population	Thailand	Northern Thailand	Laos	Northern Vietnam	Myanmar	Hong Kong	South Korea
Reference	This study	[5]	[16]	[17]	[18]	[19]	[20]
Thailand	*12.86*	13.19	13.07	12.91	12.75	13.17	12.85
Northern Thailand	0.05	*13.42*	13.40	13.20	13.09	13.36	13.07
Laos	0.05	0.09	*13.18*	13.00	13.07	13.33	13.07
Northern Thailand	0.07	0.07	− 0.01*	*12.83*	12.87	13.10	12.83
Myanmar	0.10	0.15	0.25	0.24	*12.45*	13.10	12.75
Hong Kong	0.17	0.07	0.16	0.11	0.30	*13.16*	12.65
South Korea	0.55	0.48	0.60	0.54	0.65	0.20	*11.75*

Above diagonal: Average number of pairwise differences between populations ($\hat{\varPi}$XY)

Diagonal elements: Average number of pairwise differences within population ($\hat{\varPi}$X)

Below diagonal: Corrected average pairwise difference ($\hat{\varPi}$XY-($\hat{\varPi}$X+$\hat{\varPi}$Y)/2)

*Corrected *p* value > 0.05

Pairwise F_ST_-values between population samples were relatively low and similar, indicating a close relation between populations. Higher variance was found between Southeast Asian populations (Thailand, Northern Thailand, Laos, Northern Vietnam, and Myanmar) and South Korea (F_ST_ 0.037–0.051), whereas genetic variance between Hong Kong and South Korea was low (F_ST_ 0.016). All F_ST_-values between Thailand and other population samples were significant (Table 4).

**Table 4:** Analysis of molecular variance (AMOVA) of 7 Southeast and East Asian populations (analyzed range: np 16024–576). Population pairwise F_ST_-values

Population:	Thailand	Northern Thailand	Laos	Northern Vietnam	Myanmar	Hong Kong	South Korea
Reference:	This study	[5]	[16]	[17]	[18]	[19]	[20]
Thailand	*	0.004	0.004	0.005	0.008	0.013	0.043
Northern Thailand	0.027	*	0.007	0.005	0.012	0.005	0.037
Laos	0.028	0.006	*	− 0.001	0.019	0.012	0.046
Northern Vietnam	0.015	0.012	0.598	*	0.018	0.008	0.042
Myanmar	0.000	0.000	0.000	0.000	*	0.023	0.051
Hong Kong	0.000	0.004	0.000	0.000	0.000	*	0.016
South Korea	0.000	0.000	0.000	0.000	0.000	0.000	*

Above diagonal: Population pairwise F_ST_-values

Below diagonal: *p* values (significance level = 0.05; number of permutations: 1023)

### Heteroplasmy

We observed a total of 27 point heteroplasmies at 14 different positions in 26 hair samples (12.2%) (Supplementary Table S5). In a former study including 691 hair shaft samples, the frequency of point heteroplasmy was 11.4% [21]. Based on other studies using various types of tissue material, in Southeast and East Asian populations, the percentage of samples with point heteroplasmy was calculated as follows: Northern Thailand 2.6% (blood), Laos 3.7% (blood), Hong Kong 8.5% (blood), Myanmar 8.6% (blood), South Korea 10.3% (blood and buccal swabs), and Northern Vietnam 15.0% (buccal swabs) [5, 16–20]. These values confirm that mtDNA heteroplasmy frequency is dependent on the analyzed tissue as specified in [7]. However, it has to be considered that low level heteroplasmic positions were not detected using Sanger sequencing.

## Conclusion

The sample of 213 mtDNA control region sequences will serve as a high-quality mtDNA reference for Thailand. Most of the detected haplotypes were unique within the known data and will complement the available data from Northern Thailand [5] and other Southeast Asian populations.

Recently, 960 complete mtDNA genomes from Thailand originally sequenced to investigate anthropologic questions [4] were also incorporated into the EMPOP database [14]. The increasing data of Southeast Asian mtDNA sequences is leading to a reliable forensic reference for this region.

## Electronic supplementary material

ESM 1 (DOCX 62 kb)

ESM 2 (XLSX 22 kb)

## Data Availability

All haplotypes are provided in the supplementary materials and were provided to EMPOP under the accession number EMP00699.
