# Supplementary material for: Mitochondrial DNA control region variation in a population sample from Thailand
Source: Int J Legal Med. 2020 May 1;134(5):1563–8. doi: 10.1007/s00414-020-02303-2 (PMC7417404; doi:10.1007/s00414-020-02303-2)
Supplement: Supplementary file 1 — (DOCX 62 kb) [file 414_2020_2303_MOESM1_ESM.docx]

**Table S2** PCR primers

| Name | Sequence | Amplicon length (bp) | Multiplex PCR |
| --- | --- | --- | --- |
| F15908 | 5‘ TACACCAGTCTTGTAAACC 3‘ | 525 | 1 |
| R16432 | 5‘ TGTGCGGGATATTGATTTCA 3‘ |  | 1 |
| F16450^a^ | 5‘ GCTCCGGGCCCATAACACTTG 3‘ | 549 | 1 |
| R429 | 5‘ CTGTTAAAAGTGCATACCGC 3‘ |  | 1 |
| F16268 | 5‘ CACTAGGATACCAACAAACC 3‘ | 461 | 2 |
| R159 | 5‘ AAATAATAGGATGAGGCAGGAATC 3‘ |  | 2 |
| F314^a^ | 5‘ CCGCTTCTGGCCACAGCACT 3‘ | 298 | 2 |
| R611 | 5‘ CAGTGTATTGCTTTGAGGAGG 3‘ |  | 2 |

**Table S3** Sequencing primers

| No. | Name | Sequence | Multiplex PCR |
| --- | --- | --- | --- |
| 1 | F15908 | 5‘ TACACCAGTCTTGTAAACC 3‘ | 1 |
| 2 | R16432 | 5‘ TGTGCGGGATATTGATTTCA 3‘ | 1 |
| 3 | F16450^a^ | 5‘ GCTCCGGGCCCATAACACTTG 3‘ | 1 |
| 4 | R429 | 5‘ CTGTTAAAAGTGCATACCGC 3‘ | 1 |
| 5 | F16268 | 5‘ CACTAGGATACCAACAAACC 3‘ | 2 |
| 6 | R159 | 5‘ AAATAATAGGATGAGGCAGGAATC 3‘ | 2 |
| 7 | R611 | 5‘ CAGTGTATTGCTTTGAGGAGG 3‘ | 2 |
| 8 | F15^a^ | 5‘ CACCCTATTAACCACTCACG 3‘ | 1 |
| 9 | R293 | 5‘ AATTTTTTGTTATGATGTCTGTGTGG 3‘ | 1 |
| 10 | F317 | 5‘ CTTCTGGCCACAGCACTTAAAC 3‘ | 2 |
| 11 | R599^a^ | 5‘ TTGAGGAGGTAAGCTACATAA 3‘ | 2 |
| 12 | F15971 | 5‘ TTAACTCCACCATTAGCACC 3‘ | 1 |
| 13 | R16410 | 5‘ GAGGATGGTGGTCAAGGGAC 3‘ | 1 |
| 14 | R381 | 5‘ GCTGGTGTTAGGGTTCTTTG 3‘ | 1 |
| 15 | F361^a^ | 5‘ ACAAAGAACCCTAACACCAGC 3‘ | 2 |
| 16 | F16190^a^ | 5‘ CCCCATGCTTACAAGCAAGT 3‘ | 1 |
| 17 | R16291 | 5‘ GGTAGGTTTGTTGGTATC 3‘ | 1 |
| 18 | F16363 | 5‘ CCCCATGGATGACCCCCC 3‘ | 2 |

Primers 1–7: as PCR primers (without L314); Primers 8–11: additional routine sequencing primers; Primers 12–18: additional sequencing primers in case of incomplete sequences

^a^Primers adopted from A. Brandstätter, C.T. Peterson, J.A. Irwin, S. Mpoke, D.K. Koech, W. Parson, T.J. Parsons, Mitochondrial DNA control region sequences from Nairobi (Kenya): Inferring phylogenetic parameters for the establishment of a forensic database, Int. J. Legal Med. 118 (5) (2004) 294–306. https://doi.org/10.1007/s00414-004-0466-z.

**Table S4** Haplotypes shared between the population sample from Thailand and six other East Asian populations

|  | Population: | Thailand | Northern Thailand | Laos | Northern Vietnam | Myanmar | Hong Kong | South Korea |
| --- | --- | --- | --- | --- | --- | --- | --- | --- |
|  | Reference: | This study | [5] | [16] | [17] | [18] | [19] | [20] |
|  | Number of haplotypes: | 170 | 145 | 171 | 159 | 189 | 313 | 230 |
| Haplo­type | Haplogroup |  |  |  |  |  |  |  |
| Thai070 | B5a | 6 | 3 | 5 | 4 | 4 | 4 | – |
| Thai039 | F1a | 4 | 8 | 2 | 4 | 10 | 2 | – |
| Thai107 | B4g | 3 | – | 2 | – | – | – | – |
| Thai052 | F1a1a | 2 | 7 | 4 | 5 | 26 | 2 | – |
| Thai157 | M7b1a1b | 2 | 2 | 2 | – | – | 5 | – |
| Thai069 | C | 2 | 1 | – | – | 1 | – | – |
| Thai082 | N9a2'4'5'11 | 2 | – | 1 | – | – | – | – |
| Thai153 | M20 | 2 | – | – | – | 1 | – | – |
| Thai166 | B6a1a | 1 | – | – | – | 12 | – | – |
| Thai003 | B5a1d | 1 | 1 | 3 | 1 | 2 | – | – |
| Thai015 | M7c2 | 1 | – | 1 | 1 | – | 2 | – |
| Thai065 | C4a'b'c | 1 | – | 2 | – | – | 2 | – |
| Thai096 | R9b2 | 1 | – | 1 | 2 | – | – | – |
| Thai127 | F1a1a | 1 | – | 2 | – | 1 | – | – |
| Thai142 | M24b | 1 | – | 3 | – | – | – | – |
| Thai009 | B5a | 1 | – | 1 | – | 1 | – | – |
| Thai016 | F1a1a | 1 | 2 | – | – | – | – | – |
| Thai030 | M20 | 1 | 1 | – | – | 1 | – | – |
| Thai054 | F1a1a | 1 | – | 2 | – | – | – | – |
| Thai058 | R+16189 | 1 | – | 1 | 1 | – | – | – |
| Thai067 | R9b1b | 1 | – | 2 | – | – | – | – |
| Thai077 | C4a1 | 1 | 1 | – | – | – | 1 | – |
| Thai150 | F3a1 | 1 | 2 | – | – | – | – | – |
| Thai005 | F1a | 1 | 1 | – | – | – | – | – |
| Thai010 | B5a | 1 | – | 1 | – | – | – | – |
| Thai028 | M7b1a1a3 | 1 | – | – | – | – | 1 | – |
| Thai046 | B5a1d | 1 | 1 | – | – | – | – | – |
| Thai073 | B5a | 1 | – | – | – | – | 1 | – |
| Thai078 | M | 1 | – | 1 | – | – | – | – |
| Thai080 | B4b1a+207 | 1 | 1 | – | – | – | – | – |
| Thai090 | D4j+(16286) | 1 | – | – | – | – | 1 | – |
| Thai119 | D5a3 | 1 | – | – | – | – | 1 | – |
| Thai136 | M21b2 | 1 | – | – | – | 1 | – | – |
| Thai139 | D4a | 1 | – | – | – | 1 | – | – |
| Thai145 | R9b1 | 1 | 1 | – | – | – | – | – |
| Thai152 | B4a1e | 1 | 1 | – | – | – | – | – |
| Thai160 | M | 1 | 1 | – | – | – | – | – |
| Thai168 | F1a1d | 1 | – | – | – | – | 1 | – |
| Thai173 | R9b1 | 1 | – | – | – | – | 1 | – |
| Thai195 | M | 1 | – | – | – | – | – | 1 |
| Thai197 | M72 | 1 | – | – | – | 1 | – | – |
| Thai203 | F1a1a | 1 | – | 1 | – | – | – | – |
| Thai209 | R9b1 | 1 | – | – | – | – | 1 | – |
| Thai210 | B4c2 | 1 | 1 | – | – | – | – | – |
| Number of shared haplotypes: | | 44 | 17 | 19 | 7 | 13 | 14 | 1 |
| Proportion of shared haplotypes (%): | | **25.9** | **11.7** | **11.1** | 4.4 | 6.9 | 4.5 | **0.4** |
| Number of samples with shared haplotypes: | | 59 | 35 | 37 | 18 | 62 | 25 | 1 |
| Proportion of samples with shared haplotypes (%): | | 27.7 | 18.4 | 17.3 | 9.6 | **19.0** | 6.6 | 0.4 |

**Table S5** Point heteroplasmic positions in the sample from Thailand

Sum of samples with point heteroplasmy: 26; proportion of samples: 12.2%.

Sum of point heteroplasmies: 27 (Y: 20 at 7 different positions, R: 6, W: 1).

| Sample ID | Haplogroup | Heteroplasmic variant | Nucleotide substitution |
| --- | --- | --- | --- |
| Thai040 | M | 16093Y | T → C |
| Thai041 | C | 16093Y | T → C |
| Thai053 | M9a1b1 | 16189Y | T → C |
| Thai064 | C4a1a+195 | 16093Y | T → C |
| Thai066 | R9 | 16093Y | T → C |
| Thai072 | M74a | 16093Y | T → C |
| Thai074 | N22 | 177Y | T → C |
| Thai075 | R9b2 | 16093Y | T → C |
| Thai111 | M21b2 | 16286Y | C → T |
| Thai120 | M7c | 16093Y | T → C |
| Thai122 | F3a | 234R | A → G |
| Thai130 | F1a1a | 195Y | T → C |
| Thai143 | F1a | 189R | A → G |
| Thai144 | R9 | 16093Y | T → C |
| Thai147 | R9b1 | 508R | A → G |
| Thai154 | B4+16261 | 16093Y | T → C |
| Thai159 | C | 16093Y | T → C |
| Thai161 | F1a1d | 16093Y, 16390R | 16093T → C, 16390G → A |
| Thai172 | R+16189 | 16093Y | T → C |
| Thai177 | B5a | 408W | T → A |
| Thai179 | B4a1 | 152Y | T → C |
| Thai182 | F1a1a | 16311Y | T → C |
| Thai188 | F1a1a | 200R | A → G |
| Thai190 | B5a | 16093Y | T → C |
| Thai194 | B5a | 16093Y | T → C |
| Thai215 | M21b2 | 16389R | G → A |
